# Antimicrobial Activities against Periodontopathic Bacteria of *Pittosporum tobira* and Its Active Compound

**DOI:** 10.3390/molecules19033607

**Published:** 2014-03-24

**Authors:** Jung-Hyun Oh, Yong Joon Jeong, Hyun Jung Koo, Dae Won Park, Se Chan Kang, Hoang Viet Bach Khoa, Le Ba Le, Joon Hyeong Cho, Jin-Yong Lee

**Affiliations:** 1 Department of Maxillofacial Biomedical Engineering, School of Dentistry, Kyung Hee University, Kyunghee Daero 26, Dongdaemungu, Seoul 130-701, Korea; 2 Department of Life Science, Gachon University, Seongnam Daero 1342, Seongnam, Gyeonggi-Do 461-701, Korea; 3 Institute of Oral Biology, Kyung Hee University, Kyunghee Daero 26, Dongdaemungu, Seoul 130-701, Korea; 4 Department of Biological & Environmental Science, Dongguk University, Pildongro-1gil 30, Junggu, Seoul 100-715, Korea

**Keywords:** periodonititis, *Pittosporium tobira*, natural products, R1-barrigenol, triterpene sapogenin

## Abstract

The study of medicinal plants for treatment of periodontitis is of great value to establish their efficacy as sources of new antimicrobial drugs. Five hundred and fifty eight Korean local plant extracts were screened for antibacterial activity against representative periodontopathic bacteria such as *Porphyromonas gingivalis*, *Prevotella intermedia*, and *Fusobacterium nucleatum.* Among the various medicinal plants, the alcohol extract of *Pittosporum tobira*, which significantly exhibited antibacterial effect for all tested strains, showed the highest activity in the antimicrobial assays. NMR analyses revealed that R1-barrigenol, a triterpene sapogenin, was the most effective compound in *P. tobira*. These results demonstrated that *P. tobira* possesses antimicrobial properties and would be beneficial for the prevention and treatment of periodontitis.

## 1. Introduction

Periodontitis is a common disease, with 5%–30% prevalence in the adult population [1,2]. It is a polymicrobial infection involving numerous Gram-negative pathogens embedded in a complex biofilm called dental plaque, which results in the destruction of the periodontal connective tissue and resorption of the alveolar bone [2,3]. Recent studies suggested that chronic infections, including those associated with periodontitis, increase the risk of systemic diseases such as coronary heart disease and preterm delivery of low-birth weight infants [4]. Because organisms cannot be removed from the majority of the periodontal pockets by mechanical therapy alone, antimicrobial chemotherapy may further suppress the periodontal pathogens and increase the benefits obtained by conventional mechanical treatment [2]. However, the systemic administration of antimicrobials has been reported to cause the development of multiresistant microorganisms, interbacterial transfer of resistance determinants, and side effects [5]. Moreover, in investigations of numerous systemic and local antimicrobial chemotherapeutic agents for the treatment of periodontitis, some of the antibiotics showed ineffectiveness, which may be due to the development of drug-resistant strains [2,6,7,8,9,10]. Indeed, *Porphyromonas gingivalis* and *Prevotella intermedia*, representative periodontopathogens, are resistant to many antibiotics including penicillins, cephalosphorins, and tetracyclines [11,12]. Therefore, the development of alternative antimicrobial approaches for the treatment of periodotitis is of great relevance.

For centuries, plants have been used throughout the world as drugs and remedies for various diseases, including infectious diseases [13,14]. These drugs serve as prototypes to develop more effective and less toxic medicines [15,16]. According to the WHO, medicinal plants would be the best source for obtaining a large variety of drugs [17,18]. Many plants have been used as remedies for diseases and offer biologically active compounds that possess antimicrobial properties. Thousands of constituents that can be used as sources of antimicrobial agents have been reported [19,20,21].

In this study, 558 Korean local plant extracts were screened for antibacterial activity against representative periodontopathic bacteria (*i.e.*, *P. gingivalis*, *P. intermedia*, and *Fusobacterium nucleatum*). Among these plants, 10 plant extracts were selected that had significant antibacterial effects against at least one bacterial strain. Here, we described the inhibitory effects of the selected plant extracts against the aforementioned periodontopathic bacteria. Some fractions of *Pittosporum tobira* Ait, which exhibited antibacterial effect against all the tested strains, were also evaluated to verify and isolate possible effective medicinal compounds for the treatment of periodontitis.

## 2. Results and Discussion

### 2.1. Antibacterial Activity of the Plant Extracts against Periodontopathogens

Due to increased resistance to antibiotics, antibacterial activity of natural products with a high level of safety is increasing interest [22,23]. Therefore, by employing the disc diffusion test, a total of 558 plant extracts were tested for their antibacterial activity against *P. gingivalis*, *P. intermedia*, and *F. nucleatum*. Among 558 extracts, 10 showed antibacterial activity against at least one of the tested bacteria. The plant extracts showing antibacterial effect are listed in Table 1. *P. tobira* was the only plant that demonstrated antibacterial activity against all the three tested bacteria, and was selected for further studies.

**Table 1:** Antibacterial activity of Korean local plant extracts against representative periodontopathic bacteria.

Family	Scientific Name	Part	Activity ^a^		
			Pg	Pi	Fn
Primulaceae	*Lysimachia mauritiana* Lam.	Whole plant	-	+	+++
Betulaceae	*Alnus firma* S. et Z.	Leaf	++	-	-
Betulaceae	*Carpinus laxiflora* Bl.	Leaf	+	-	-
Taxaceae	*Torreya nucifera* S. et Z.	Fruit	-	-	+++
Fabaceae	*Albizzia julibrissin* Durazz	Leaf	-	-	++
Fabaceae	*Albizzia julibrissin* Durazz	Fruit	++	-	+++
Euphorbiaceae	*Sapium japonicum* Pax et Hoffm	Leaf	+	-	-
Pittosporaceae	*Pittosporum tobira* Ait.	Leaf	+	+	++
Lardizabalaceae	*Akebia quinata* Decne.	Fruit	++	-	-
Lauraceae	*Litsea japonica* Juss.	Fruit	-	-	+++
Erythromycin			+++	+++	+++

^a^ Activity: Diameter of inhibition zone <9 mm, -; 9–11 mm, +; 12–14 mm, ++; >15 mm, +++.

### 2.2. Antibacterial Effect of P. tobira against Periodontopathogens

Using the 96-well plate dilution method, the antibacterial activity of *P. tobira* against the three major periodontopathogens *P. gingivalis*, *P. intermedia*, and *F. nucleatum* was determined. Among the other fractions, the EtOAc fraction exhibited the strongest antibacterial effect on the tested strains and MIC was determined to be 200 μg/mL for all the bacteria tested (Table 2). However, *P. tobira* extract did not show inhibitory effect for all the strain tested in the concentration range (>800 μg/mL).

**Table 2:** Susceptibility of representative periodontopathogens to the fractions obtained from *Pittosporum tobira* ethanolic extract (PE) partition between immiscible solvents.

Bacterial strains	MICs (μg/mL)							
	EtOH80%	*n*-Hexane	MC	EtOAc	*n*-BuOH	DW	Erythromycin	
Pg	>800	400	800	200	>800	800	<0.02	
Pi	>800	200	800	200	>800	400	<0.02	
Fn	>800	200	800	200	>800	400	<0.02	

Pg: Porphyromona gingivalis, Pi: Prevotella intermedia, and Fn: Fusobacterium nucleatum.

### 2.3. Chemistry

**Figure 1:**
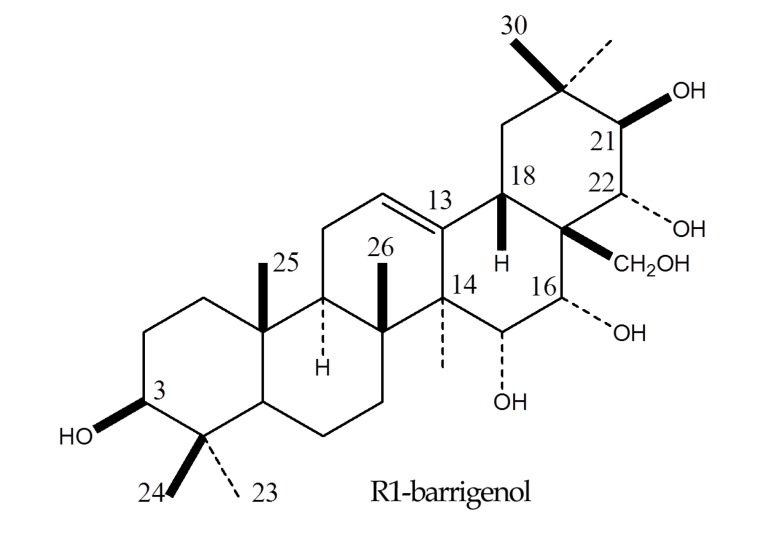
Structure of the active compound from *P. tobira*.

The EtOAc fraction was fractionated by chromatography over silica gel eluting with EtOAc, followed by increasing concentrations of methanol to yield five fractions. Successive column chromatographic purification of the second fraction (Fr.2) led to the isolation and characterization of a kind of sapogenin. The isolated compound was identified as R1-barrigenol (Figure 1) by comparison of its NMR spectrum with those of authentic samples and reference data [24].

*P. tobira* Ait. (Pittosporaceae) is a small, slender, evergreen shrub that grows in Japan, China, and Korea. Some research interested on the chemical composition of *P. tobira* including triterpenoids, saponins, and carotenoids [24,25] and a saponin mixture from its leaves showed that this plant possesses antibiotic activity [24]. However, there are only a few reports on the biological properties of the compounds contained in the plant.

We therefore identified various active fractions and isolated the major components of *P. tobira*. A sample subfractionated with EtOAc potently inhibited periodontopathic bacteria growth. Our detailed phytochemical investigation revealed that the major antibacterial molecule in *P. tobira* is R1-barrigenol, which was characterized by NMR.

### 2.4. Antibacterial Effect of R1-Barrigenol against Periodontopathogens

**Figure 2:**
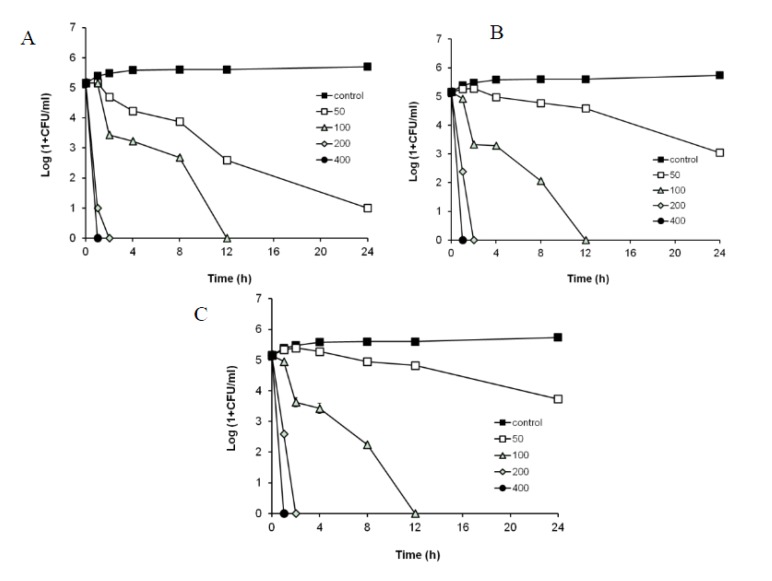
Time-kill curve of R1-barrigenolfrom *P. tobira* against *P. gingivalis* (**A**), *P. intermedia* (**B**), and *F. nucleatum* (**C**) growth. R1-barrigenol(50–400 μg/mL) completely kills all the strains after 12 h.

According to the CLSI guidelines, the minimum bactericidal concentration (MBC) is defined as the minimum concentration needed to kill ≥99.9% (≥3 log10) of the viable organisms after a 24-h incubation relative to the starting inoculum [26,27]. In time-kill experiments using a starting inoculum of 10^5^ CFU/mL, the bactericidal effect of the R1-barrigenol separated from *P. tobira* was observed at concentrations of 50–400 μg/mL. The MBC of the R1-barrigenol was determined to be 100 μg/mL for these three bacteria (Figure 2). Generally, it is known that only one anti-periodontitis agent is effective against a specific strain of periodontopatic bacteria.

The cellular toxic effects of the compounds contained in *P. tobira* on NIH/3T3 mouse embryonic fibroblast cells were assessed using the MTT assay. The results showed that the R1-barrigenol did not affect the cell viability and was not cytotoxic to NIH/3T3 at the concentrations used (data not shown). The results suggest that R1-barrigenol is not toxic to normal cells, selectively kills the bacteria.

## 3. Experimental

### 3.1. Plant Materials

A total of 558 plant extracts [80% ethanol (EtOH) extracts] (Table 1) were obtained from the Jeju Biodiversity Research Institute (Seogwipo, Korea). Voucher specimens have been deposited at the Department of Life Science, Gachon University (Seongnam, Korea). The plant extracts were dissolved in dimethylsulfoxide (DMSO) and used as samples for antibacterial activity screening tests. The leaves of *P. tobira* were collected in Jeju of Korea (same first collected region), and a voucher specimen (No. JBR-111) has been deposited in Department of Life Science, Gachon University.

### 3.2. Antibacterial Activity Screening: Disc Diffusion Test

The periodontopathic bacterial strains *P. gingivalis* ATCC33277, *P. intermedia* ATCC 25611, and *F. nucleatum* subsp. *nucleatum* ATCC 23726 were grown in half-strength brain heart infusion (BHI) broth (Difco Laboratories, Detroit, MI, USA) supplemented with 5 mg/mL yeast extract, 5 µg/mL hemin, and 1 μg/mL vitamin K_1_ (BHI-HK). The bacteria grown at 37 °C anaerobically (85% N_2_, 10% H_2_, and 5% CO_2_). The disc diffusion method [28] was used to screen the antimicrobial activity. The *in vitro* antimicrobial activity was screened by using half-strength BHI agar supplemented with 5% defibrinated sheep blood. The optical density of the bacterial inocula was adjusted to 0.1 at 600 nm (0.5 McFarland standard). Each bacterial inoculum suspension (100 μL) was swabbed uniformly on a blood agar plate, and the plate was allowed to dry for 5 min. Different concentrations of extracts (2.5, 5 and 10 mg/mL) were loaded at 20 μL onto a 6-mm sterile disc (50, 100 and 200 μg/disc, respectively). The loaded disc was placed on the surface of the medium, the compound was allowed to diffuse for 5 min, and the plates were incubated at 37 °C for 48 h. At the end of the incubation, the inhibition zones formed around the disc were measured with a transparent ruler in millimeter units. This experiment was performed in triplicate.

### 3.3. Extraction and Solvent Partitions of P. tobira

**Scheme 1:**
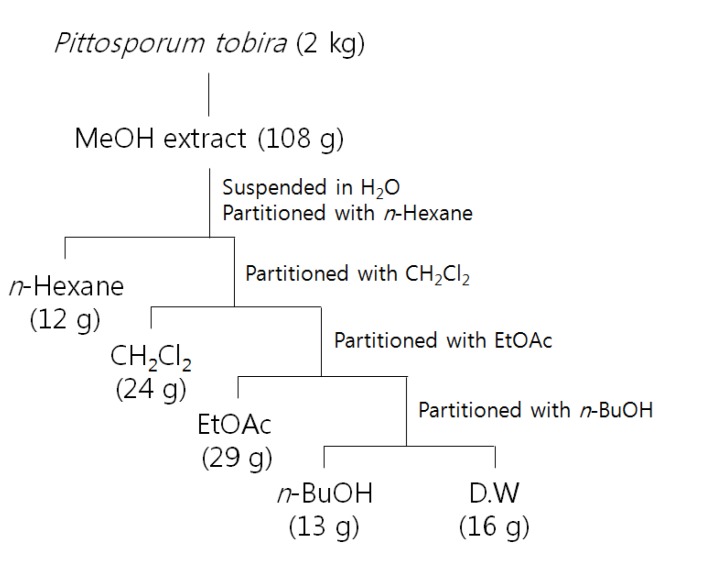
Extraction and solvent partitions from MeOH extract of *P. tobira*.

*P. tobira*, which showed antibacterial activity against all the tested bacteria in the disc diffusion test, was selected for further studies. The dried and powdered plant (2.0 kg) of *P. tobira* was percolated three times with MeOH at room temperature. The filtrates were combined and evaporated to dryness under vacuum. The dried filtrate (108 g) was suspended in distilled water (D.W.; 800 mL) and extracted with *n*-Hexane (800 mL × 3; 12 g), CH_2_Cl_2_ (800 mL × 3; 24 g), EtOAc (800 mL × 3; 29 g), *n*-BuOH (800 mL × 3; 13 g), and D.W (16 g), successively (Scheme 1).

### 3.4. Determination of the Minimal Inhibitory Concentrations (MIC) and Minimal Bactericidal Concentration (MBC)

MIC was determined with 96-well plate microdilution method. Briefly, each bacterial strain was grown for 24 h anaerobically and inoculated into a final volume of 100 μL of new half-strength BHI broth containing 2-fold serial dilutions of *P. tobira* fractions. The final optical density of the bacterial cells was adjusted to 0.1 at 600 nm in 100 μL of mixture. The mixture was cultured anaerobically at 37 °C for 48 h and the bacterial growth was evaluated via measurement of the optical density at 600 nm. The lowest concentration at which no growth (OD_600nm_ ≤ 0.1) was observed was defined as MIC (μg/mL).

Time-kill experiments were performed to determine MBC of the R1-barrigenol separated from *P. tobira* for the bacteria in brucella broth containing hemin and vitamin K1 according to CLSI guidelines. The R1-barrigenol was tested at concentrations of 1/4 to 2 MIC of the *P. tobira* EtOAc fraction (200 μg/mL). Bacterial inocula of 10^5^ CFU/mL were incubated with the R1-barrigenol. Aliquots were removed from the bacterial cultures at 0, 4, 8, 12, and 24 h, and plated on BHI blood agar for 24 h. Viable cells were enumerated by counting the number of CFU.

### 3.5. Cell Culture and Cellular Toxicity Assay

NIH/3T3 cells were purchased from American Type Culture Collection (Manassas, VA, USA) and were grown in DMEM medium (Gibco BRL, Grand Island, NY, USA) containing 10% bovine calf serum (Gibco BRL) and antibiotics (100 U/mL penicillin and 100 mg/mL streptomycin; Gibco BRL) at 37 °C in a humidified atmosphere containing 5% CO_2_. Cellular toxicity was measured by quantitative colorimetric assay by using the 3-(4,5-dimethylthiazol-2-yl)-2,5-diphenyltetrazolium bromide (MTT), which shows the mitochondrial activity of living cells. Cells were cultured in a 96-well microplate until confluent, then were treated with or without compound of *P. tobira* for 12 h, and subsequently incubated with MTT for 4 h. The extent of the reduction of MTT to formazan within the cells was quantified by measuring the optical density at 540 nm using a Molecular Device microplate reader (Ramsey, MN, USA).

### 3.6. Isolation of the Compounds

**Scheme 2:**
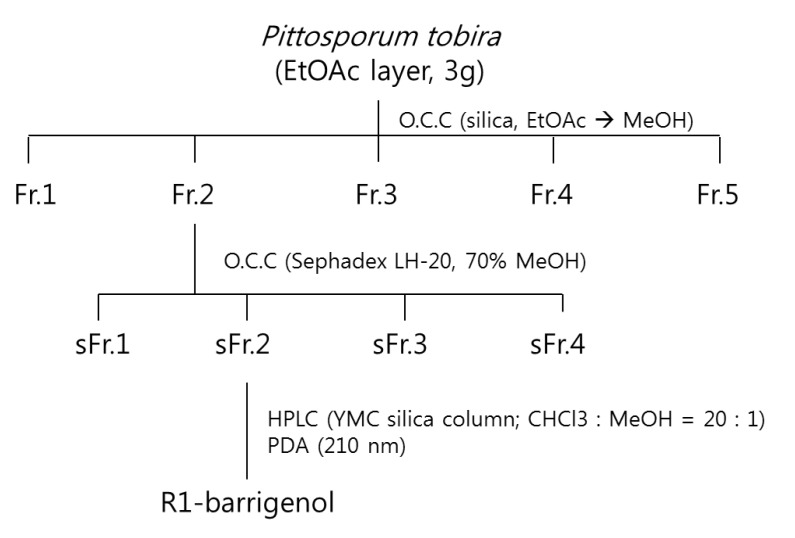
Isolation of R1-barrigenol from EtOAc extract of *P. tobira*.

The EtOAc fraction (3 g from 29 g) was fractionated by adsorption chromatography over silica gel eluting with EtOAc followed by increasing concentrations of methanol to yield five fractions (Fr.1–5). Fr.2 (442 mg) was firstly injected into a Sephadex LH-20 column eluting with 70% MeOH to yield four fractions (sFr.1–4). sFr.2 (38 mg) was purified by semi-preparative HPLC on a YMC silica (5 μm, 250 × 10 mm ID) column (mobile phase, CHCl_3_/MeOH [20:1]; flow rate 2 mL/min; UV detection, 210 nm], affording compound (Scheme 2).

*R1-Barrigenol*. ^1^H-NMR (300 MHz, pyridine-*d*_5_): *δ* 0.98, 1.05, 1.11, 1.24, 1.35, 1.40, 1.88 (3H each, s, Me × 7), 3.78 (1H, br s, H-3), 3.79, 4.13 (2H, ABq, *J* = 10.4 Hz, H-28), 4.43 (1H, d, *J* = 4.5Hz, H-15), 4.62 (1H, d, *J* = 9.6 Hz, H-22), 4.83 (1H, d. *J* = 9.6 Hz, H-21), 4.97 (1H, d, *J* = 4.5 Hz, H-16), and 5.54 (1H, H-12); ^13^C-NMR (75 MHz, pyridine-*d*_5_): *δ* 15.9 (C-25), 16.5 (C-24), 17.5 (C-26), 19.1 (C-6), 19.3 (C-30), 20.8 (C-27), 23.9 (C-11), 28.1 (C-2), 28.6 (C-23), 30.5 (C-29), 36.2 (C-20), 36.7 (C-7), 37.2 (C-10), 39.1 (C-1), 39.3 (C-4), 41.4 (C-8), 42.0 (C-18), 47.1 (C-9), 47.3 (C-14), 47.8 (C-19), 48.1 (C-17), 55.6 (C-5), 67.4 (C-15), 67.7 (C-28), 72.3 (C-16), 77.1 (C-22), 78.0 (C-3), 78.3 (C-21), 124.4 (C-12), 144.6 (C-13).

## 4. Conclusions

Periodontitis is a destructive inflammatory disease that leads to the loss of tooth support. It is initiated in the oral microbial biofilm constituted by Gram-negative anaerobic bacteria, including *P. gingivalis*, *P. intermedia*, and *F. nucleatum*. Our findings provide evidence that R1-barrigenol, which is a triterpene sapogenin from *P. tobira* successfully inhibits periodontal bacteria strains. Although a detailed study of the action mechanism of this molecule remains to be elucidated, it could be more beneficial than traditional antibiotics as a potential agent for prevention and/or treatment of periodontitis.
